# Psoriasis: Pathogenesis, Comorbidities, and Therapy Updated

**DOI:** 10.3390/ijms22062979

**Published:** 2021-03-15

**Authors:** Naoko Kanda

**Affiliations:** Department of Dermatology, Nippon Medical School Chiba Hokusoh Hospital, Inzai, Chiba 2701694, Japan; n-kanda@nms.ac.jp; Tel.: +81-476-99-1111; Fax: +81-476-99-1909

**Keywords:** oxidative stress, antimicrobial peptide, innate immune cell, nutrient, UBA domain containing 1, indoleamine 2,3-dioxygenase 2, fibroblast, collagen disease, metabolic disease, pruritus, gut dysbiosis, treatment

## Abstract

Psoriasis is a chronic inflammatory skin disease characterized by IL-17-dominant abnormal innate and acquired immunity, and the hyperproliferation and aberrant differentiation of epidermal keratinocytes, and comorbid arthritis or cardiometabolic diseases. This Special Issue presented updated information on pathogenesis, comorbidities, and therapy of psoriasis. The pathogenesis of psoriasis may involve the dysfunction of indoleamine 2,3-dioxygenase 2 or of UBA domain containing 1-mediated regulation of CARD14/CARMA2*sh*. The blood cells of psoriasis patients showed the enhanced oxidative stress/autophagy flux and decreased 20S proteasome activity. Elafin, clusterin, or selenoprotein P may act as biomarkers for psoriasis and comorbid metabolic diseases. The proteomic profile of psoriasis lesions showed the dysfunction of dermal fibroblasts; up-regulation of proinflammatory factors and signal transduction or down-regulation of structural molecules. The skin inflammation in psoriasis may populate certain gut bacteria, such as *Staphylococcus aureus* and *Streptococcus danieliae,* which worsen the skin inflammation in turn. The psoriasis-associated pruritus may be caused by immune, nervous, or vascular mechanisms. In addition to current oral treatments and biologics, a new treatment option for psoriasis is now being developed, such as retinoic-acid-receptor-related orphan nuclear receptor γt inhibitors, IL-36 receptor antagonist, or aryl hydrocarbon receptor agonist. Antimicrobial peptides and innate immune cells, involved in the pathogenesis of psoriasis, may be novel therapeutic targets. The pathomechanisms and responses to drugs in collagen diseases are partially shared with and partially different from those in psoriasis. Certain nutrients can exacerbate or regulate the progress of psoriasis. The articles in this Special Issue will encourage attractive approaches to psoriasis by future researchers.

Psoriasis is a chronic inflammatory skin disease characterized by abnormal innate and acquired immunity and the hyperproliferation and aberrant differentiation of epidermal keratinocytes, and comorbid arthritis or cardiometabolic diseases. In this Special Issue, we have published seven research articles and seven reviews on pathogenesis, comorbidities, and therapy of psoriasis.

Mazzone et al. noticed the scaffold protein CARD14/CARMA2*sh,* the prominent isoform of CARMA2 expressed in the human skin, for the control of psoriasis [1]. CARMA2 controls signal transduction pathways converging on the activation of NF-κB, and mutations in *CARMA2* are associated with familial psoriasis. CARMA2*sh* interacts with the UBA domain containing 1 (UBAC1), the non-catalytic subunit of the E3 ubiquitin–protein ligase KPC complex. UBAC1 promotes K63-linked ubiquitination of TANK, an adaptor protein of CARMA2*sh*, and negatively regulates the Toll-like receptor (TLR)3-induced expression of genes, *IL-8*, *S100A8/9/12*, *CCL20* in keratinocytes, while the abrogation of UBAC1 exacerbates these responses. UBAC1-mediated regulation of CARMA2*sh*-TANK complex may be potential therapeutic targets for the treatment of psoriasis.

It is reported that enhanced oxidative stress is associated with the severity of psoriasis. Karabowicz et al. investigated the intensity of oxidative stress and the expression and activity of the proteasomal system as well as autophagy, responsible for the degradation of oxidatively modified proteins in the blood cells of patients with psoriasis [2]. The caspase-like, trypsin-like, and chymotrypsin-like activity of the 20S proteasome in lymphocytes, erythrocytes, and granulocytes was lower in patients with psoriasis compared to controls. The autophagy flux was increased in lymphocytes and erythrocytes of patients with psoriasis compared to controls. The levels of 4-hydroxy-2,3-trans-nonenal (4-HNE), 4-HNE-protein adducts, and carbonylated proteins reflecting lipid peroxidation were higher in lymphocytes, erythrocytes, and granulocytes of patients with psoriasis compared to controls. These results indicate a proteostatic imbalance in the blood cells of patients with psoriasis. The enhancement of autophagy, favoring NF-κB activation, may increase the production of proinflammatory cytokines in patients with psoriasis. The use of antioxidant compounds in the treatment of psoriasis may support the metabolic homeostasis in the blood cells of patients.

Patients with psoriasis are frequently associated with metabolic syndromes, such as obesity, glucose intolerance, dyslipidemia, or hypertension. Clusterin (apolipoprotein J), synthesized in most mammalian cells as a consequence of cellular stress, has both pro- and anti-inflammatory effects. Elafin, also known as SKALP (skin-derived anti-leukoprotease), is a serine protease inhibitor produced by keratinocytes, and its expression is increased in proinflammatory microenvironments. Holmannova et al. examined the frequency of metabolic syndromes, serum elafin and clusterin levels, and their relationship in patients with psoriasis, compared to healthy controls [3]. Serum clusterin and elafin levels were higher in patients with psoriasis than in controls. In psoriasis patients with metabolic syndromes, the level of clusterin was lower than in psoriasis patients without. Clusterin may play a protective role, such as binding to membrane attack complex of complements, and prevent cytolysis or inhibit NF-κB activity. Its higher level in patients with psoriasis may represent a compensatory protective mechanism. However, the presence of metabolic syndromes in patients with psoriasis may accelerate the endocytosis and redistribution and reduce the serum clusterin levels. Elafin is a potent inhibitor of proteolytic enzymes, such as neutrophil elastase. The higher level of elafin in patients with psoriasis may reflect a compensatory protective mechanism. The other biomarkers for metabolic syndromes associated with psoriasis should further be identified.

Indoleamine 2,3-dioxygenase 1 (IDO1), an enzyme that metabolizes tryptophan to kynurenine, is known to suppress immune responses. It is reported that the defect of IDO1 induction is associated with psoriasis. Fujii et al. noticed the IDO2, an isoform of IDO1, expressed in dendritic cells (DCs) and monocytes [4]. *IDO2* knockout mice showed exaggerated imiquimod-induced psoriasis-like dermatitis compared to wild-type mice, with enhanced TNF-α, IL-23p19, and IL-17A expression. The results indicate that IDO2 may downregulate the IL-17 signaling pathway. Whether patients with psoriasis show the defect of IDO2 induction should further be studied.

Gęgotek et al. assessed the changes in the proteomic profile of dermal fibroblasts in the psoriasis lesions and discussed their consequence [5]. The proteomic results indicate that fibroblast dysfunction arises from the upregulation of proinflammatory factors (S100A8/9, NF-κB, TNF-α) and antioxidant proteins (glutaredoxin, thioredoxin, Nrf2), as well as those involved in signal transduction (serine/threonine–protein phosphatase 2A, channel protein 4) and proteolytic processes (calpain, 26S proteasome). Moreover, downregulated proteins in fibroblasts of psoriasis lesions are responsible for the transcription/translation processes, glycolysis/adenosine triphosphate synthesis and structural molecules (β-catenin, importin-8, galectin-3). These changes in fibroblasts may directly affect intercellular signaling and promote the hyperproliferation of epidermal cells. A better manipulation of the proteomic changes in fibroblasts could develop new pharmacotherapies for psoriasis. Dermal fibroblasts may become another therapeutic target for psoriasis.

Selenoprotein P, a member of hepatokines, has anti-inflammatory properties by neutralizing reactive oxygen species (ROS), and its serum level is elevated in patients with various metabolic diseases. Baran et al. examined serum selenoprotein P levels in patients with psoriasis and healthy volunteers [6]. Selenoprotein P concentration was higher in patients with psoriasis than in controls. In patients with severe psoriasis, selenoprotein P level before treatment was increased compared with controls and decreased after treatment. Selenoprotein P concentration positively correlated with C-reactive protein value and platelet count and negatively with red blood cell count. Selenoprotein P may be a novel indicator of inflammation and metabolic complications in patients with psoriasis, especially with the severe form or with concomitant obesity. Further larger sample-sized studies should investigate the significance of selenoprotein P in psoriasis and concomitant metabolic syndromes.

The skin and gut dysbiosis of patients with psoriasis have been reported. Okada et al. examined the gut microbiome of keratinocyte-specific caspase-1 transgenic (Kcasp1Tg) mice by analyzing the 16S rRNA gene [7]. *Staphylococcus aureus* and *Streptococcus danieliae* were abundant in Kcasp1Tg mouse fecal microbiome. These dominant bacteria, as well as recessive control bacteria, were orally administered to antibiotic-treated wild-type mice, and psoriasis-like skin inflammation was induced by topical imiquimod. *Staphylococcus aureus* and *Streptococcus danieliae*-administrated groups showed the exacerbated skin lesions with elevated levels of TNF-α, IL-17A, IL-17 F, and IL-22, compared to those with recessive control bacteria. The results suggest a vicious cycle between skin inflammation and gut microbiome: skin inflammation populates certain gut bacteria, which worsen the skin inflammation in turn. Not only treating the skin lesion but also treating the gut microbiome, such as probiotics treatment, could be the future key therapy for psoriasis.

Approximately 60–90% of patients with psoriasis have pruritus, and it deteriorates their quality of life. Komiya et al. reviewed the features of pruritus in psoriasis and discussed the mechanisms by which a variety of itch mediators induce or aggravate itch in psoriasis [8]. Various types of neuropeptides, substance P, calcitonin gene-related peptide, neuropeptide Y, act on nerves to induce itch. Opioid receptors, their ligands, and the transient receptor potential family may modulate the degree of itch. Nerve growth factor and IL-31 promote the growth of sensory nerves and potentiate the itch sensation. Various immune cells, such as mast cells or T cells, secrete cytokines mediating itch, such as IL-31, thymic stromal lymphopoietin or IL-2. Gamma-amino butyric acid derived from macrophages stimulates various immune cells to secrete other pruritogenic mediators. The corticotropin-releasing hormone and α-melanocyte-stimulating hormone may be related to psychological stress-induced pruritus. Vascular endothelial growth factor, prostaglandin E_2_, endothelin-1, or cell adhesion molecules may induce itch by enhancing angiogenesis and vascular permeability, and recruiting immune cells to lesional sites. Clinical trials are ongoing for candidates of antipruritics, such as inhibitors of the neurokinin-1 receptor, tropomyosin-receptor kinase A, or phosphodiesterase 4.

Tokuyama M and Mabuchi T reviewed new insights on the pathogenesis of psoriasis in relation to DCs, Langerhans cells, macrophages, the signal transducer and activator of transcription 3 pathway, and aryl hydrocarbon receptor in vascular endothelial cells [9]. They summarized currently available oral treatments and biologics, and described a new treatment option, including retinoic-acid-receptor-related orphan nuclear receptor γt inhibitors, IL-36 receptor antagonist, Janus kinase inhibitors, tyrosine kinase 2 inhibitor, sphingosine 1-phosphate receptor 1 agonist, Rho-associated kinase 2 inhibitor, and aryl hydrocarbon receptor agonist.

Antimicrobial peptides (AMPs), such as β-defensin, S100, and cathelicidin, activate the innate immune system and induce inflammation. Takahashi T and Yamasaki K reviewed the roles of AMPs in the pathogenesis of psoriasis [10]. AMPs are involved in the Köbner phenomenon in psoriasis; microinjuries or scratches induce keratinocytes to release damage-associated molecular patterns (DAMPs), such as self-DNA and RNA, and AMPs complexed with these DAMPs bind to the receptors TLR7/8/9 in plasmacytoid DCs or keratinocytes, and promote their secretion of type I interferons. Myeloid DCs, activated by the type I interferons, secrete TNF-α, IL-12, or IL-23, which potentiates the proliferation and survival of Th17 cells. Neutrophil extracellular traps, complexes of self-DNA and AMPs including LL-37 released from neutrophils, act on Th17 and Th1 cells, modulate cellular signaling and enhance their cytokine production. LL-37 may act as an autoantigen in psoriatic arthritis. The blocking or degradation of AMPs may be a novel treatment for psoriasis.

Recent findings demonstrated that innate immune cells contribute to the development of psoriasis. Sato et al. discussed the roles of innate immunity in psoriasis [11]. Innate lymphoid cells, γδ T cells, natural killer T cells, and natural killer cells are activated in psoriasis, contributing to disease pathology through IL-17-dependent and -independent mechanisms. The transplantation of these cell types into mouse models successfully induced psoriasis phenotype, while the induction of psoriasis was reduced in mouse models lacking these cell types. In patients with psoriasis, the number of natural cytotoxicity receptor-positive group 3 innate lymphoid cells in peripheral blood decreased after treatment, suggesting that these cells are markers of psoriasis severity.

Pleńkowska et al. demonstrated that the development and exacerbation of psoriasis are related to the increased production of ROS (hydrogen peroxide, superoxide radical) and reactive nitrogen species (nitric oxide radical), and decreased concentration/activity of antioxidants (superoxide dismutase, catalase, glutathione peroxidases, glutathione, ascorbic acid, β-tocopherol, and α-carotene) [12]. Oxidative stress in psoriasis leads to the activation of many signaling pathways, including NF-κB and mitogen-activated protein kinases, and consequently, to the activation of Th1 and Th17 cells, hyperproliferation of keratinocytes, and vascular permeability through lipid peroxidation. Though the remarkable therapeutic effects of biologics have been reported for psoriasis, they are not effective for all patients. Treatment aimed at oxidative stress, such as antioxidant supplementation, may become an add-on therapy for those patients.

Yamamoto T discussed several connective tissue diseases and related diseases, such as systemic lupus erythematosus, rheumatoid arthritis (RA), dermatomyositis, systemic sclerosis, granulomatous diseases from the viewpoint of their coexistence with psoriasis [13]. In addition to the genetic background, shared pathogenesis, including innate immunity, neutrophil extracellular traps, and type I interferons, as well as acquired immunity, such as Th17 cells, may play a significant role in both psoriasis and connective tissue diseases. On the other hand, there are definite differences between psoriasis and connective tissue diseases in their pathomechanisms and responses to drugs. IL-17 blockers are effective for psoriasis while not for RA. By contrast, anti-IL-6 receptor antibody is effective for RA, while not for psoriasis. Therefore, caution is necessary when considering whether the administered drug for one disease is effective or not for another disease.

Finally, Kanda et al. discussed the relationship between nutrition and psoriasis [14]. Nutrition influences the development and progress of psoriasis and its comorbidities, such as obesity, diabetes, cardiovascular diseases, or inflammatory bowel diseases. Saturated fatty acids, simple sugars, red meat, or alcohol exacerbate psoriasis via the activation of inflammasome, TNF-α/IL-23/IL-17 pathway, ROS, prostanoids/leukotrienes, gut dysbiosis or suppression of regulatory T cells, while *n*-3 polyunsaturated fatty acids, vitamin D, vitamin B12, short-chain fatty acids, selenium, genistein, dietary fibers or probiotics ameliorate psoriasis via the suppression of inflammatory pathways above or induction of regulatory T cells. The manipulation of the disease-regulatory effects of nutrients or food may be useful for the management of psoriasis.

All 14 articles have brought a new wind in the research of psoriasis from individual viewpoints and will encourage attractive approaches by future researchers. There exist differences in immune systems between human psoriasis and psoriatic model mice. Some paradoxical reactions to biologics have been identified in clinical sites. The model mice for psoriasis and psoriatic arthritis have not been completely established. These problems will hopefully be solved by future enriched research from both basic and clinical viewpoints.
